# Effect of anthelminthic treatment on helminth infection and related anaemia among school-age children in northwestern Ethiopia

**DOI:** 10.1186/s12879-016-1956-6

**Published:** 2016-10-28

**Authors:** Yonas Yimam, Abraham Degarege, Berhanu Erko

**Affiliations:** 1 Department of Biology, Faculty of Natural and Computational Sciences, Woldia University, P.o. box. 400, Woldia, Ethiopia; 2 Department of Epidemiology, Robert Stemple College of Public Health, Florida International University, Miami, USA; 3 Aklilu Lemma Institute of Pathobiology, Addis Ababa University, P.O. Box 1176, Addis Ababa, Ethiopia

**Keywords:** Helminths, Anthelminthic treatment, Haemoglobin, Anaemia, Ethiopia

## Abstract

**Background:**

Information about improvements in the health status of population at-risk of helminth infection after anthelminthic treatment helps to evaluate the effectiveness of the large scale deworming program. The objectives of this study were to assess the impact of anthelminthic treatment on the prevalence and intensity of intestinal helminth infection, haemoglobin level and prevalence of anaemia among school-age children.

**Methods:**

A total of 403 children attending Tikur Wuha Elementary School in Jiga, northwestern Ethiopia were enrolled in this study between February and March 2011. Formol-ether concentration and Kato-Katz methods were used to examine stool for intestinal helminth infections at baseline and one month after anthelminthic treatment. Haemoglobin level was measured using Hemocue machine at baseline and one month after anthelminthic treatment.

**Results:**

Out of 403 school children examined, 15.4 % were anaemic and 58.3 % were infected with intestinal helminths at baseline. Hookworms (46.9 %), *Schistosoma mansoni* (24.6 %), *Ascaris lumbricoides* (4.2 %) and *Trichuris trichiura* (1.7 %) infections were common. The odds of anaemia was higher among children infected with helminths (adjusted odds ratio (aOR) = 3.83, 95 % CI = 1.92, 7.62) especially in those infected with hookworm (aOR = 2.42, 95 % CI = 1.34, 4.39) or *S. mansoni* (aOR = 2.67, 95 % CI = 1.46, 4.88) and two or more helminth species (aOR = 7.31, 95 % CI = 3.27, 16.35) than those uninfected with intestinal helminths at baseline. Significant reduction in prevalence of helminth infection (77.0 %) and increment in mean haemoglobin level (+3.65 g/l) of children infected with helminths was observed one month after anthelminthic treatment. The increase in haemoglobin level after anthelminthic treatment was significantly positively associated with the age, but negatively associated with the haemoglobin level at baseline. The change in mean haemoglobin level was significantly higher among undernourished than normal children. Percent reduction in the prevalence of anaemia among children infected with helminths was 25.4 % after anthelminthic treatment.

**Conclusions:**

The present study provides evidence that anthelminthic treatment of school-age children infected with intestinal helminth can improve haemoglobin level in addition to reducing the prevalence and intensity of helminth infections one month after treatment. This suggests that deworming of children may benefit the health of children in sub-Sharan Africa where hookworm and *S. mansoni* infections are prevalent.

## Background

Neglected tropical diseases (NTDs) are heterogeneous group of disease due to viral, bacteria or parasite infections such as soil transmitted helminth (STH) and *Schistosoma mansoni* [1]. NTDs pose public health problem for over one billion people worldwide particularly in the developing countries [1]. NTDs are responsible for over 48 million disability-adjusted life years lost each year [2].

Today a scale-up of mass drug administration program is underway in various parts of Africa, Asia and South-America to control NTDs such as schistosomiasis and STH by 2020 [1, 3]. The program involves regular administration of anthelminthic drugs to at-risk populations, usually without prior diagnosis of every infected individual [4]. Accordingly, most children living in sub-Saharan Africa where helminth infection are endemic are treated in mass with anthelminthic drugs [5]. Several pharmaceutical companies donate millions of drugs to ensure the success of the program [6]. Given the large amount of money invested, it should be clear whether mass or targeted anthelminthic treatment can lead to improvement in health and development in such a substantive way. However, it is not clear whether the deworming programs brought changes in the prevalence of helminth infection and related problems such as anaemia and undernutrition as anticipated.

Intestinal helminth infections are very common in Ethiopia particularly in school-age children and can reach up to 83.3 % in some regions [7]. As a result, the country launches a mass deworming program in November 2015 to treat at risk children in schools for STH and *Schistosoma* infection [8]. Several cross-sectional studies confirmed association of intestinal helminth infection with low haemoglobin level and high prevalence of anaemia among children in the country [9–11]. These associations suggest that deworming may help to reduce the prevalence of anaemia. However, due to the cross-sectional nature of the study designs, it is possible that the associations might have been caused by other socioeconomic factors. Hence, interventional studies are required to confirm whether the mass treatment program can improve the haemoglobin level and reduce the prevalence of helminth infection. Information about improvements in the health status of the at-risk population after anthelminthic treatment will help to evaluate the effectiveness of the large scale deworming program. Thus, the current study was conducted to evaluate the impact of treatment with anthelminthic drugs in reducing the prevalence and intensity of helminth infection and related anaemia among school-age children in Tikur Wuha Elementary School, northwestern Ethiopia.

## Methods

### Study area and population

The study was longitudinal and involved 403 children attending Tikur Wuha Elementary School, northwestern Ethiopia in February and March, 2011. The school is situated in Jiga District, approximately 390 km from Addis Ababa. The area has an elevation of 1917 m above sea level with an average temperature and rainfall of 18.5 °C and 134.35 mm, respectively. More than 95 % of the population in the area composed of the Amhara ethnic group and agriculture and trade are the main income source for majority of the community. Most of the people in the district live in houses with iron sheets/grass roof, mud/wooden wall and mud floor. Intestinal helminth infections are common among communities in the area (health facility records). Children of age 5 to 15 years attending the school during the specified period and who apparently looked healthy were included in the study. Children included in the study were homogenous in terms of their areas of residence, housing conditions, life style, ethnicity and families’ socio-economic status.

### Nutrition assessment

Height and weight of all the children was measured in bare foot and lightly-worn clothes to the nearest 100 cm and 1 kg, respectively at baseline and one month after anthelminthic treatment in children who were infected with helminth at baseline. Then, Z-values for weight-for-age (for only children aged between 5 and 10 years) (WAZ), body mass index-for-age (BAZ) and height-for-age (HAZ) were calculated [12]. Children with Z- values of BAZ, WAZ and HAZ > −2 were grouped as well-nourished and those with Z-values of either BAZ or WAZ or HAZ < −2 were grouped as under-nourished [13].

### Haemoglobin measurement

Approximately 10 μl of finger prick blood was collected from all children and haemoglobin level was determined using hemocue machine (Hemocue HB 201, Anghelom, Sweden) at baseline. Haemoglobin level of children infected with intestinal helminths and treated with anthelminthic drug was re-measured four weeks after treatment following similar procedure. The haemoglobin level measured was used to assess the anaemic status of the children following the WHO guidelines [14]. The WHO cut-off of haemoglobin level used for determining anaemia was 115.0 g/l for children 5–11 years; 120.0 g/l for children 12–14 years; 120.0 g/l for female children of 15 years of age and 130.0 g/l for female children with 15 years of age [14].

### Microscopic examination of stool for intestinal helminths

Approximately 5 g of fresh stool specimens was collected from each children and processed using the Kato-Katz (2 slides per individuals) and concentration techniques at baseline and one month after anthelminthic treatment [15]. Only children who were infected with intestinal helminth at baseline and treated were re-examined one month after anthelminthic treatment. Qualitative examination for all of the helminth species and simultaneous quantification in the case of hookworm infection from the Kato-Katz slides was made on the spot. However, egg count for other helminth species from the Kato-Katz slides and processing of samples in the formal-ether concentration method was done at Aklilu Lemma Institute of Pathobiology, Addis Ababa University (ALIPB).

### Anthelminthic treatment

Only children who were found positive for helminth infection were treated with anthelminthic drugs. Children who were infected with STHs were treated with 400 mg albendazole while those who were infected with *H. nana*, *T. saginata* and *S. mansoni* infection were treated with praziquantel in appropriate doses (40 mg/kg body weight). Children who were infected with STHs and *H. nana*, *T. saginata* or *S. mansoni* were treated with both 400 mg albendazole and praziquantel (40 mg/kg body weight).

### Statistical analysis

Data were computerized using Excel 2007 and analyzed using STATA version 11 (Stata Corporation, College Station, Texas, USA). Paired *t*-test was used to check if the difference in the mean egg counts and mean haemoglobin levels before and after anthelminthic treatment was significant. Unpaired *t*-test was used to check if the difference in the mean haemoglobin levels changes after anthelminthic treatment was significant between males *vs* females, children with ages 5–10 years *vs* 11*–*15 years, undernourished *vs* normal children. Z-test was used to compare the prevalence of intestinal helminth infection and prevalence of anemia before and after anthelminthic treatment. Multiple logistic regression analysis was used to assess factors associated with the prevalence of helminth infection and prevalence of anaemia. Multiple linear regression analysis was used to assess factors associated with intensity of helminth infection and haemoglobin level. Values were considered significant whenever *P-*value was less than 5 %.

## Results

### Prevalence and intensity of helminth infection

A total of 403 children (mean age in years ± SD, 11.42 ± 2.42) were examined for intestinal helminth infection and 58.3 %, 46.9 %, 24.6 %, 4.2 %, 1.7 % and 2.0 % of these were infected with at least one helminth species, hookworm, *S. mansoni*, *A. lumbricoides*, *T. trichiura* and *H. nana* or *T. saginata* infections, respectively. Out of 235 children infected with intestinal helminths, 31.9 % were infected with two or three different helminth species. *S. mansoni* infection was positively associated with hookworm (adjusted odds ratio (aOR) = 1.89, 95 % CI = 1.16, 3.07) or *A. lumbricoides* infection (aOR = 4.11, 95 % CI = 1.40, 12.09). The odds of *S. mansoni* infection was higher among males than females (aOR = 1.70, 95 % CI = 1.05, 2.75). However, infections with *A. lumbricoides, T. trichiura* and hookworm were comparable between males and females or between children of age 5–10 years and 11–15 years (Table 1). Majority of the infections were light and some of the infections were moderate (Table 1). Only one individual had heavy intensity *S. mansoni* infection. The mean egg per gram of *A. lumbricoides*, *T. trichiura*, hookworm and *S. mansoni* infections were 1205.6, 634.3, 191.5 and 72.5, respectively.

**Table 1:** Prevalence (%) and intensity of intestinal helminths infection among 403 school-age children in Tikur Wuha Elementary School, northwestern Ethiopia, 2011

Variables	Number examined	Hookworm	*S. mansoni*	*A. lumbricoides*	*T. trichiura*	Others^a^	Any helminth
Age (years)							
5-10	146	50.0	21.3	4.8	2.1	4.1	54.8
11-15	257	45.1	26.5	3.9	1.6	0.8	60.3
Total	403	46.9	24.6	4.2	1.7	2.0	58.3
Sex							
Female	216	47.2	19.9	4.6	1.9	2.3	55.6
Male	187	46.5	29.9	3.7	1.6	1.6	61.5
Intensity							
Light	290	100	79.8	94.1	8.6		
Moderate	21	-	19.2	5.9	14.3		
Heavy	1	-	1.0	-	-		
Mean egg per gram		191.5	72.48	1205.6	634.3		

Others^a^  = *T. saginata* or *H. nana*

### Impact of anthelminthic treatment on prevalence and intensity of helminth infection

Out of 235 children infected with intestinal helminths and treated with anthelmithic drugs, 86.4 % cured. However, 7.2 %, 5.5 %, 0.8 % and 0.8 % of the children treated with anthelmithic drug (235) continued to excrete eggs of hookworm, *T. trichiura*, *A. lumbricoides* and *S. mansoni*, respectively one month after anthelmithic treatment. The prevalence of hookworm, *S. mansoni A. lumbricoides* and *T. trichiura* infections significantly reduced one month after anthelminthic treatment (Table 2). The mean egg counts of hookworm and *S. mansoni* significantly reduced one month after anthelminthic treatment (Table 2). However, the reduction in mean egg count of *A. lumbricoides* and *T. trichiura* after treatment was not significant.

**Table 2:** Effect of anthelminthic treatment on the prevalence (%) and mean egg count of intestinal helminths among 403 school-age children in Tikur Wuha Elementary School, northwestern Ethiopia, 2011

Helminth species	Before treatment (*n* = 403)		After treatment (*n* = 235)		Percent reduction	
	Prevalence	MEPG^a^	Prevalence	MEPG^a^	Prevalence	MEPG^a^
Hookworm	46.9	89.8	7.2	14.8	84.7	83.5
*S. mansoni*	24.6	17.8	5.5	3.3	77.6	81.5
*A. lumbricoides*	4.2	50.9	0.8	12.6	81.0	75.2
*T. trichiura*	1.7	11.0	0.8	3.1	52.9	71.8
Any helminth	58.3		13.6		76.7	

MEPG^a^ = mean egg per gram

Note: All percent reduction values are significant at *p* = 0.01 except MEPG reductions in *A. lumbricoides* and *T. trichiura*

The odds of being cure from intestinal helminth infection was reduced with an increase in the intensity of hookworm infection (aOR = 0.988, 95 % CI = 0.98, 0.997) but increased with an increase in the intensity of *S. mansoni* infection at baseline (aOR = 1.03, 95 % CI = 1.00009, 1.06). However, the odds of cure rate was not associated with the age and nutritional status of children, and multiplicity of infection before treatment in a multivariable logistic regression model (data not shown).

### Impact of intestinal helminth infection on haemoglobin level and prevalence of anaemia

Out of 403 children examined (mean haemglobin = 129.5 g/l), 15.14 % were anaemic. The mean haemoglobin level was significantly lower among children infected with helminths (adjusted regression coefficient (β) = −5.80, 95 % CI = −8.23, −3.38) especially in those infected with hookworm (β = −3.98, 95 % CI = −6.40, −1.55), *S. mansoni* (β = −4.66, 95 % CI = −7.56, −1.76) and two or more helminth species (β = −9.19, 95 % CI = −12.65, −5.73) than those who were not infected with intestinal helminths. The mean haemoglobin level significantly decrease with an increase in the intensity of hookworm infection (Table 3). The mean haemoglobin level was lower among children who were undernourished than normal ones (β = −3.46, 95 % CI = −5.85, −1.60). The odds of anaemia before anthelminthic treatment was also significantly higher among children infected with helminths (aOR = 3.83, 95 % CI = 1.92, 7.62) especially in those infected with hookworm (aOR = 2.42, 95 % CI = 1.34, 4.39) or *S. mansoni* (aOR = 2.67, 95 % CI = 1.46, 4.88) and two or more helminth species (aOR = 7.31, 95 % CI = 3.27, 16.35) than those who were not infected with intestinal helminths. The odds of anaemia significantly increase with an increase in the intensity of hookworm infection (aOR = 1.003, 95 % CI = 1.001, 1.004). However, the mean haemoglobin level and odds of anaemia were similar when compared between children infected with *A.lumbricoides* or *T. trichiura* and those uninfected with intestinal helminths.

**Table 3:** Factors affecting mean haemoglobin levels (g/l) and prevalence of anaemia among 403 school-age children at baseline in Tikur Wuha Elementary School, northwestern Ethiopia, 2011

Explanatory variables	Mean haemoglobin level β^a^ (95 % CI)	Prevalence of anaemia aOR^b^ (95 % CI)
Age (continuous)	1.08 [0.59, 1.57]	1.13 [0.99, 1.28]
Males *vs* females	−0.92 [−3.29, 1.45]	0.89 [0.50, 1.59]
Undernourished *vs* normal	−3.49 [−5.86, −1.11]	1.10 [0.62, 1.96]
Infection		
Not infected	Ref.	Ref.
Hookworm	−3.98 [−6.40, −1.55]	2.42 [1.34, 4.39]
*S. mansoni*	−4.66 [−7.56, −1.66]	2.67 [1.46, 4.88]
*A. lumbricoides*	−4.13 [−10.37, 2.11]	0.99 [0.25, 3.87]
*T. trichiura*	3.06 [−6.24, 12.36]	0.71 [0.08, 6.25]
Multiple	−9.19 [−12.65, −5.73]	7.31 [3.27, 16.35]
Any helminth	−5.80 [−8.23, −3.38]	3.83 [1.92, 7.62]
Egg intensity		
Hookworm	−0.02 [−0.04, −0.003]	1.003 [1.001, 1.004]
*S. mansoni*	−0.01 [−0.03, 0.04]	1.00 [0.99, 1.005]
*A. lumbricoides*	−0.003 [−0.02, 0.03]	1.00 [0.99, 1.002]
*T. trichiura*	−0.01 [−0.03, 0.003]	1.00 [0.998, 1.00]

β^a^ (regression coefficient): based on multiple linear regression analysis adjusted for age, gender, nutritional status and multiple helminth infection

aOR^b^(adjusted odds ratio): based on multiple logistic regression analysis adjusted for age, gender, nutritional status and multiple helminth infection

### Impact of anthelminthic treatment on haemoglobin level and prevalence of anaemia

The mean haemoglobin levels of children infected with intestinal helminths significantly increased (+3.65 g/l) one month after anthelminthic treatment (*p* < 0.001). The changes in haemoglobin level after anthelminthic treatment increased with an increase in the age (β = 1.06, 95 % CI = 0.26, 1.87), but decreased with an increase in the haemoglobin level at base line (β = −0.19, 95 % CI = −0.35, −0.02). The change in mean haemoglobin level was also higher among children who were undernourished than normal ones (β = 2.54, 95 % CI = 0.45, 4.62). However, the changes in haemogobin level was not associated with the intensity of helminth infections and sex of children.

Prevalence of anaemia among children infected with helminth (21.3 %) decreased one month after anthelminthic treatment (16.1 %). However, the difference was not statically significant. The reduction in the prevalence of anaemia after anthelminthic treatment was significantly higher in females (percent reduction = 32.2 %) than in males (percent reduction = 15.5 %) (*p* < 0.01). However, the percent reduction in the prevalence of anaemia was similar in children with varying age groups, nutritional status and number of helminth species co-infecting the host (Table 4).

**Table 4:** Effect of anthelminthic treatment on mean haemoglobin level (g/l) and prevalence of anaemia (%) among school children in Tikur Wuha Elementary School, northwestern Ethiopia, 2011

Parameters	Number examined	Before treatment	After treatment	Mean haemoglobin increase/percent anaemia decrease	P*-value
Mean haemoglobin	235	127.2	130.8	3.7	<0.01
Age in years					
5–10	80	125.1	127.2	2.1	<0.01
11–15	155	128.2	132.7	4.5	<0.01
P**-value				0.031	
Sex					
Female	120	126.0	129.7	3.7	<0.01
Male	115	128.3	132.0	3.7	<0.01
P**-value				0.975	
Infection					
Hookworm	189	126.8	130.7	3.8	<0.01
*S. mansoni*	99	125.9	128.5	2.6	<0.01
*A. lumbricoides*	17	124.2	125.4	1.2	0.507
*T. trichiura*		130.3	130.9	0.6	0.386
Single	160	128.7	133.0	4.3	<0.01
Multiple	75	124.0	126.2	2.3	0.01
Nutrition					
Normal	215	129.1	131.3	2.2	<0.01
Undernourished	188	125.1	130.3	5.2	<0.01
P**-value				<0.01	
Prevalence of anaemia	235	21.3	16.1	25.4	0.148
Age in years					
5–10	80	13.8	11.3	18.1	0.633
11–15	155	25.5	18.3	28.2	0.125
P**-value				0.089	
Sex					
Female	120	25.8	17.5	32.2	0.119
Male	115	16.8	14.2	15.5	0.586
P**-value				<0.01	
Infection status					
Hookworm	189	21.7	14.3	34.1	0.061
*S. mansoni*	99	26.3	24.2	8.0	0.744
*A. lumbricoides*	17	17.7	17.7	0.0	1.00
*T. trichiura*	7	14.3	14.3	0.0	1.00
Single	160	17.5	13.2	24.6	0.286
Multiple	75	29.3	22.7	22.5	0.357
Nutrition status					
Normal	215	19.7	16.2	17.8	0.487
Undernourished	188	18.5	15.9	14.1	0.248
P**-value				0.359	

*p*-values*: based on paired *t*-test (mean haemoglobin differences between before and post treatment) and z-test of proportion (changes in the prevalence of anaemia before and post treatment)

*p*-values**: based on unpaired *t*-test (compared the magnitude of changes in haemoglobin level between two groups) and z-test of proportion (compared the magnitude of changes in the prevalence of anaemia between two groups)

## Discussion

This study confirmed that infections with intestinal helminths particularly hookworm, *S. mansoni* or two or more intestinal helminth species simultaneously are associated with an increased risk of anaemia. Fortunately, anthelminthic treatment resulted in significant reduction in prevalence of intestinal helminth infection and significant increase in haemoglobin levels one month after treatment.

Intestinal helminth infection can directly or indirectly affect haemoglobin level in blood, which may lead to iron deficiency anaemia [16–18]. For example, hookworm release anticoagulants, ingest blood, and damage the intestinal wall causing bleeding [16], while *S. mansoni* causes splenic sequestration, autoimmune haemolysis and extra-corporeal loss of iron [17]. On the other hand, *A. lumbricoides* affect haemoglobin level indirectly by reducing appetite and nutrition uptake or causing mal-adsorption in the intestine [18]. Considering differences in the means by which hookworm, *S. mansoni*, *A. lumbricoides* and *T. trichiura* affect blood iron level [16–18], these parasites are expected to have additive effect on haemoglobin level when they co-exist. Indeed, the extent of reduction in haemoglobin level and odds of anaemia among children infected with helminth in the current study increased as the number of helminth species infecting the host increases.

In agreement with the current finding, a systematic review of intervention studies estimated an increase mean haemoglobin level of +1.89 g/l and 2.37 g/l after treatment of individuals with albendazole and combined albendazole and praziquanel, respectively [19]. This suggests that deworming can increase haemoglobin by enhancing iron status by preventing helminth infection related blood loss [19, 20]. However, the amount of gains in haemoglobin level after anthelminthic treatment were not consistent among studies [19, 20]. Previous studies were heterogeneous in the type of drugs used for treating helminth infection, in techniques used for examining infection and measuring haemoglobin level, nature of the study population, prevalence and intensity of helminth infection and length of follow up period. These may have contributed to the differences in the magnitude of haemoglobin change after anthelminthic treatment.

Increase in the age of children, low haemoglobin level and low nutrition status before anthelminthic treatment were significant predictors of better mean haemoglobin gains after treatment. This finding could be due to the increased intensity and prevalence of multiple infections in those with low haemoglobin level at baseline. The reduction of heavy worm burden due to the anthelminthic drug could consequently lead to a marked increase in mean haemoglobin level one-month post-treatment. The finding of a high haemoglobin gains after treatment among children who were undernourished during baseline survey could be because of the fact that the impact of some parasite species like *T. trichiura* and *A. lumbricoides* on haemoglobin level is indirect through affecting nutrition status of the host [18]. Hence, deworming can increase haemoglobin by preventing helminth infection related undernutrition.

The change in prevalence of intestinal helminth (s) infection after anthelminthic treatment was higher in males and children with double or triple intestinal helminth infections before treatment. Children infected with two or more different intestinal helminth species were treated with praziquantel and albendazole when helminth species were *S. mansoni/T. saginata* and soil-transmitted helminths (STHs). Although albendazole is effective for treating STHs, it could have an effect against *S. mansoni/T. saginata* too and the same holds true for praziquantel. As a result, clearance of helminth will be facilitated better in children having multiple infections and treated with the two drugs simultaneously. However, the reason for the increased clearance of intestinal helminth in males compared to females is not clear. Perhaps, immunological or other factors could contribute to increased clearance of helminths in males.

To the best of our knowledge, this is the first study in Ethiopia which evaluated changes in haemoglobin level and prevalence of anaemia after anthelminthic treatment. As the country recently launches a national deworming program of school-age children, the current finding could be used as a testimonial to support the importance of the program in improving the health of the children in the country. The strength of the present study included homogeneity of the study population and the use of concentration and Kato-Katz methods for checking helminth infection. However, lack of data on helminth infection status and haemoglobin level changes in children uninfected with helminth at baseline and untreated with anthelminthic drugs made the study inadequate to mimic the potential impact of mass treatment, where treatment is given irrespective of underlying infection status. Moreover, lack of placebo-control treated group for making comparison with the drug treated group limited the study to be certain that the findings were solely due to the intervention and not to any other factors. Chance and bias due to unmeasured confounders may partly contribute to the differences observed. For example, children may got treatment for other infections such as malaria and the socioeconomic status of the parents might have been improved, which could potentially increase haemoglobin level and decrease iron deficiency anaemia [21]. However, the children involved in this study were apparently health looking at baseline and during the follow up survey, hence the chance of *Plasmodium* infection during the survey was minimal. In addition, that the length of follow up period was short strengths the view that the changes were unlikely to be due to socioeconomic improvement. On top of that, observation of significant decrease in the prevalence and intensity of helminth infection after anthelminthic treatment supports that the observed change in haemoglobin level was due to the treatment.

## Conclusions

The present study provides evidence that anthelminthic treatment of school-age children infected with helminth can improve haemoglobin level and anaemia in addition to reducing the prevalence and intensity of helminth infections one month after treatment. The magnitude of haemoglobin changes varied with the age, baseline haemoglobin level and nutrition status of the children. This confirms that deworming may benefit the health of children in sub-Sharan Africa where hookworm and *S. mansoni* infections are prevalent.
